# Understanding the wide geographic range of a clonal perennial grass: plasticity versus local adaptation

**DOI:** 10.1093/aobpla/plv141

**Published:** 2015-12-07

**Authors:** Yanjie Liu, Lirong Zhang, Xingliang Xu, Haishan Niu

**Affiliations:** 1 College of Resources and Environment, University of Chinese Academy of Sciences, No. 19A Yuquan Road, Beijing 100049, China; 2 Ecology, Department of Biology, University of Konstanz, Universitätsstrasse 10, Konstanz D-78457, Germany; 3 Key Laboratory of Alpine Ecology and Biodiversity, Institute of Tibetan Plateau Research of Chinese Academy of Sciences, No. 16 Lincui Road, Beijing 100101, China; 4 Key Laboratory and Ecosystem Network Observation and Modelling, Institute of Geographic Sciences and Natural Resources Research, Chinese Academy of Sciences, No. 11A Datun Road, Beijing 100101, China

**Keywords:** δ^13^C, ecotype, genetic differentiation, *Leymus chinensis*, phenotypic plasticity, species distribution, water use efficiency

## Abstract

Both phenotypic plasticity and local adaptation may allow widely distributed plant species to either acclimate or adapt to environmental heterogeneity. Given the typically low genetic variation of clonal plants across their habitats, phenotypic plasticity may be the primary adaptive strategy allowing them to thrive across a wide range of habitats. In this study, the mechanism supporting the widespread distribution of the clonal plant *Leymus chinensis* was determined, i.e. phenotypic plasticity or local specialization in water use efficiency (WUE; reflected by foliar δ^13^C). To test whether plasticity is required for the species to thrive in different habitats, samples were collected across its distribution in the Mongolian steppe, and a controlled watering experiment was conducted with two populations at two different sites. Five populations were also transplanted from different sites into a control environment, and the foliar δ^13^C was compared between the control and original habitats, to test for local specialization in WUE. Results demonstrated decreased foliar δ^13^C with increasing precipitation during controlled watering experiments, with divergent responses between the two populations assessed. Change in foliar δ^13^C (−3.69 ‰) due to water addition was comparable to fluctuations of foliar δ^13^C observed *in situ* (−4.83 ‰). Foliar δ^13^C differed by −0.91 ‰ between two transplanted populations; however, this difference was not apparent between the two populations when growing in their original habitats. Findings provide evidence that local adaptation affects foliar δ^13^C much less than phenotypic plasticity. Thus, plasticity in WUE is more important than local adaptation in allowing the clonal plant *L. chinensis* to occupy a wide range of habitats in the Mongolian steppe.

## Abstract (precis)

Both phenotypic plasticity and local adaptation may allow widely distributed plant species to either acclimate or adapt to environmental heterogeneity. Given the typically low genetic variation of clonal plants across their habitats, phenotypic plasticity may be the primary adaptive strategy allowing them to thrive across a wide range of habitats. In this study, we used field investigation and controlled experiments to test this. We found that plasticity in water use efficiency (reflected by foliar δ^13^C) is more important than local adaptation in allowing the studied clonal plants to occupy a wide range of habitats.

## Introduction

A major question in ecology and evolution is why plants are distributed across a wide range of environmental conditions. Phenotypic plasticity and local adaptation are two complementary mechanisms that allow plants to adjust to environmental heterogeneity. Phenotypic plasticity is the ability of a single genotype to produce different phenotypes in response to environmental variation (Bradshaw 1965; Sultan 1987; Lortie and Aarssen 1996; Murren *et al*. 2015). Several empirical studies have suggested that species can adapt to diverse habitats via phenotypic plasticity (Sultan 2001; Ayrinhac *et al*. 2004; Hulme 2008). However, the capacity of individual genotypes to perform well across all habitats is often limited (DeWitt *et al*. 1998). Geographic variation can lead to the evolution of different adaptations to the local environment, and thus generate ecotypic differentiation in important functional traits (Kawecki and Ebert 2004; Savolainen *et al*. 2007). Therefore, widespread plant species are often characterized by both phenotypic plasticity and local specialization to particular environmental conditions (Van Tienderen 1990; Joshi *et al*. 2001), though the relative contribution of these strategies to their distributions may be species specific.

*Leymus chinensis* is a perennial rhizomatous clonal C_3_ grass, widely distributed across eastern areas of the Eurasian steppe (Liu *et al*. 2007; Chen and Wang 2009; Wang *et al*. 2011). The species has a very broad distribution in China as a dominant or co-dominant plant (Liu *et al*. 2007). As an economically and ecologically important grass species, the question as to why *L. chinensis* can thrive across such a large range of arid and semi-arid regions has received considerable attention in recent years. For example, several studies have assessed the impacts of large-scale climatic variables on responses ranging from variation in population density, plant height, leaf size, biomass and biomass allocation to anatomical and physiological plasticity (Wang and Gao 2003; Wang *et al*. 2003, 2011). However, the relative importance of plasticity and local adaptation in water use efficiency (WUE) to support the persistence of *L. chinensis* across variable environments has not been extensively studied. As water is typically the most limiting factor for growth and community productivity across a plant's distribution (Huxman *et al*. 2004; Chen *et al*. 2005), selecting an indicator related to WUE can help to better understand whether, and to what extent, phenotypic plasticity versus local adaptation influences the distribution of a species thriving in arid and semi-arid regions. Foliar δ^13^C is an important indicator of plant long-term WUE (Farquhar *et al*. 1982; Ehleringer 1993; Saurer *et al*. 2004; Liu *et al*. 2013) and, therefore, can be used to assess water adaptation strategies of *L. chinensis* across its distribution.

In this study, we determined whether plasticity or local specialization in WUE (reflected by foliar δ^13^C) supports the widespread distribution of the clonal plant *L. chinensis*. Given the typically low genetic variation of clonal plants across their habitats, phenotypic plasticity may be the most important adaptive strategy allowing them to thrive (Parker *et al*. 2003; Geng *et al*. 2007; Riis *et al*. 2010). However, Chen and Wang (2009) reported that two ecotypes of *L. chinensis* occur across its distribution, with divergent anatomies and physiologies. Therefore, local specialization in WUE of this clonal plant may also be apparent across its distribution. We hypothesized that both plasticity and local specialization in WUE contribute to the widespread distribution of this clonal species, and that plasticity is the primary adaptive mechanism. If so, foliar δ^13^C of this clonal plant would be rapidly changed in response to changes in habitat water conditions, potentially to the extent of variations in foliar δ^13^C apparent across its entire distribution. Additionally, local specialization in foliar δ^13^C would also be apparent among populations dominating different habitats.

## Methods

### Response of *L. chinensis* foliar δ^13^C to natural environments in the Mongolian steppe

In order to investigate natural variations in *L. chinensis* foliar δ^13^C, a large-scale field study in the Mongolian steppe was conducted; half of the study region was located in Mongolia and half in Inner Mongolia, China. The study area extended ∼15° longitude, from 106.4° to 121.7° longitude, and 6° latitude, from 43.72° to 49.43° latitude. The climate was predominantly arid and semi-arid continental, and mean annual precipitation (MAP) ranged from ∼190 to 400 mm. The main vegetation types distributed from west to east across the study area were desert steppe, typical steppe and meadow steppe.

Mature leaves of *L. chinensis* were collected from 24 sites across west to east Mongolia in 2008, and 18 sites across west to east Inner Mongolia in 2011 **[see** **Supporting Information—Table S1****]**. The majority of sampling sites were located far from cities and were considered to be under natural conditions, without significant human influence. In order to adequately sample environmental heterogeneity and avoid biased sampling of, for example, one specific microhabitat, the topography of each site was assessed, and samples collected systematically from lower to higher elevations along the hillside aspect at each site. The sampling area at each site was 50 m^2^ (1 × 50 m). In the 2008 field campaign, samples were collected across the 50-m^2^ sampling area and pooled at each site, resulting in one sample for each site assessed. In the 2011 field campaign, five samples were systematically collected from five 1 × 1 m plots, extending in 10-m intervals from a low to high position along the hillside aspect at each site, resulting in five samples per site assessed in 2011. For each sample, all mature leaves (except withered leaves) of five to eight randomly chosen *L. chinensis* individuals were collected and pooled for later analyses.

### Plasticity in *L. chinensis* foliar δ^13^C in two controlled watering experiments

In order to assess changes in *L. chinensis* foliar δ^13^C due to precipitation, two controlled watering experiments were conducted at two sites in Inner Mongolia in 2011: a desert steppe site MDLT and a typical steppe site ABGQ **[see** **Supporting Information—Table S1****]**. These experiments formed part of a larger experiment for other purposes (see Liu *et al*. 2014). At each site, 18 plots (1 × 1 m) were established, and 6 levels of supplementary watering (0, 20, 40, 60, 80 and 100 % of local MAP [MDLT, ∼215 mm; ABGQ, ∼263 mm]) were assigned to triplicate plots. The total amount of water per treatment level was divided into five equal parts, and evenly applied five times during the growing season, from 18 June to 7 August. During each watering event, water was applied evenly to each plot using a portable 1-m^2^ plot boundary, constructed of mild steel, and a watering can, used as a simple rainfall simulator. Some soil was piled up around the metal frame to minimize any leakage from the plot. In late August 2011, after one season of watering, mature *L. chinensis* leaves (except withered leaves) were collected from both sites for subsequent analyses.

### Variation among different *L. chinensis* populations in a transplant experiment

In order to assess potential adaptive variations in foliar δ^13^C among different populations of *L. chinensis* originating from various locations, four populations of *L. chinensis* were transplanted to a field station, and the foliar δ^13^C compared with plants from the source populations. In 2009, we chose four sites from west to east Inner Mongolia: MDLT, XLHT, XWQ and HH **[see** **Supporting Information—Table S1****]**. *Leymus chinensis* ramets were collected from each site and transplanted to a field experimental site located at the Maodeng Grassland Ecology Research Station (MDMC), in central Inner Mongolia. Twelve plots (2 × 2 m) were established, though only three were used in this study; the remainder were used for the study of Liu *et al*. (2013). Ten *L. chinensis* ramets were transplanted into each plot and were watered until the end of the growing season (late September 2009) to ensure survival. Transplants were not watered during 2010, and in late September 2010, all mature leaves (except withered leaves) of the transplanted ramets were collected across plots, and pooled to make one sample for each plots. Individual ramets of local *L. chinensis* populations were also randomly selected for mature leaf collection, resulting in the overall collection of samples from five *L. chinensis* populations. In order to investigate variations in foliar δ^13^C among these five populations under natural conditions, comparative sampling was also performed in their respective original collection sites in 2011.

### Carbon isotope measurement

All leaves were immediately microwaved (500 W, 2 min) after collection to ensure deactivation of plant enzymes, and subsequently air-dried. Once back in the laboratory, samples were further dried in a drying oven at 65 °C for 48 h. All collected plant material was ground to a homogenous powder using a ball mill (MM200; Retsch, Haan, Germany).

Aliquots of ∼2.5 mg of plant material were weighed into tin capsules for foliar δ^13^C measurement. For samples collected during the transplant experiment, foliar δ^13^C was measured using continuous-flow gas isotope ratio mass spectrometry (CF-IRMS) with Vario PYRO Cube (IsoPrime100; Isoprime Ltd, Stockport, UK) at the Institute of Environment and Sustainable Development in Agriculture, CAAS, China. Reproducibility was high, with the standard deviation of repeated measurements <0.20 ‰. For all other samples assessed in this study, foliar δ^13^C was measured using CF-IRMS with Flash EA1112 and the interface Conflo III (MAT 253; Finnigan MAT, Bremen, Germany) at the Institute of Geographic Sciences and Natural Resources Research, Chinese Academy of Sciences, China. Reproducibility was high, with the standard deviation of repeated measurements <0.15 ‰. Vienna Pee Dee Belemnite was used as the reference standard for carbon isotopic analyses.

### Data analysis

All statistical analyses were performed using R v.3.1.3 (R Development Core Team 2015). For both *in situ* investigations and controlled watering experiments, the correlation between foliar δ^13^C and precipitation was examined using linear regression. This correlation reflects the plasticity of foliar δ^13^C to changes in precipitation. To test for local specialization in foliar δ^13^C plasticity between the two populations of the controlled watering experiment, differences between the regression slopes of the two populations were tested using the smatr package (Warton *et al*. 2012). For the transplantation experiment, one-way analysis of variance (ANOVA) was used to test for differences between *L. chinensis* populations originating from various locations. Tukey's honest significant differences (Tukey's HSD) analysis was used to examine significant differences highlighted by ANOVAs. One-way ANOVA was also used to test for differences between respective transplanted (data from 2010) and field (data from 2011) populations.

## Results

**Figure 1.**
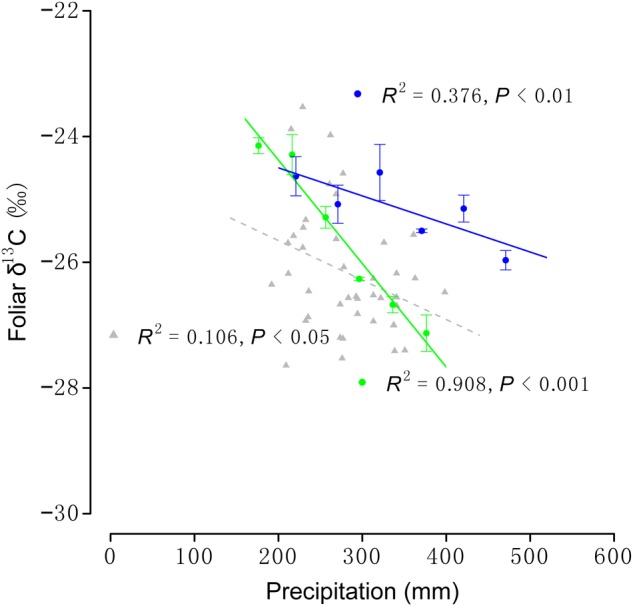
The response of *L. chinensis* foliar δ^13^C to precipitation during field investigations and controlled watering experiments. Grey triangles and dashed line represent field investigation data. Circles and solid lines represent data from the controlled watering experiment conducted in MDLT (green) and ABGQ (blue). Each point represents mean (±SE) foliar δ^13^C (*n* = 5).

In the field investigation, *L. chinensis* foliar δ^13^C decreased with increasing precipitation across its distribution (Fig. 1; *F*_1,40_ = 4.755, *R*^2^ = 0.106, *P* < 0.05). Comparable responses were observed for *L. chinensis* populations at MDLT (*F*_1,16_ = 158.3, *R*^2^ = 0.908, *P* < 0.001) and ABGQ (Fig. 1; *F*_1,16_ = 9.655, *R*^2^ = 0.376, *P* < 0.01). Data obtained from transects in the Mongolian steppe showed a range in *L. chinensis* foliar δ^13^C of −4.83 ‰, from −27.92 to −23.09 ‰. Water addition induced change in foliar δ^13^C values of −3.69 ‰, from −27.43 to −23.74 ‰, for the two populations examined during the controlled watering experiments. Significant differences between the two population's responses to water treatments were observed (Fig. 1; *χ*^2^ = 13.32, df = 1, *P* < 0.001).

**Figure 2.**
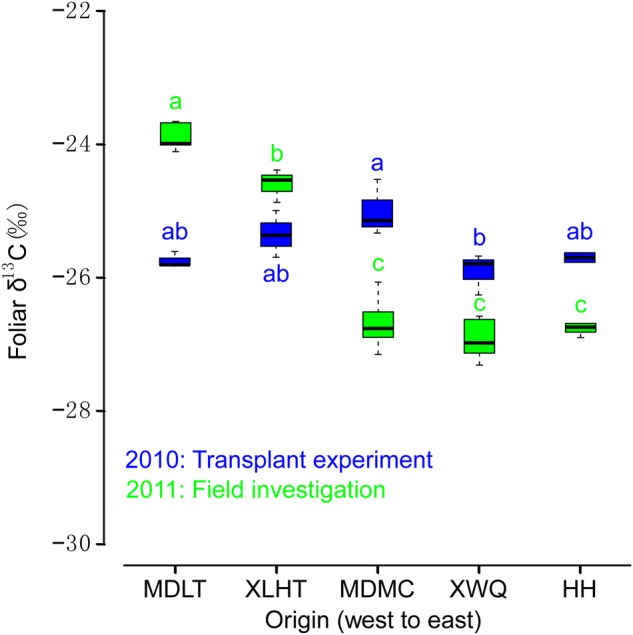
Foliar δ^13^C values of five *L. chinensis* populations from different geographical regions assessed during the 2010 transplant experiment (blue) and 2011 field investigation (green). Box plots show median (solid line) values. Lowercase letters indicate homogeneous subsets identified by ANOVA and Tukey's HSD analyses (*P* < 0.05).

In the transplant experiment, a significant difference in *L. chinensis* foliar δ^13^C was observed in relation to original collection site (*F*_4,9_ = 4.230, *P* < 0.05), i.e. there was local specialization in foliar δ^13^C among populations (Fig. 2). However, Tukey's HSD analysis revealed that statistically significant differences were apparent only between populations originating from MDMC and XWQ, the former exhibiting significantly higher foliar δ^13^C; no other significant differences were apparent between populations (Fig. 2). Mean foliar δ^13^C differed by −0.91 ‰ between MDMC and XWQ populations. In the field investigation, significant differences in foliar δ^13^C were observed for the five populations assessed in 2011 (*F*_4,27_ = 142.4, *P* < 0.001). Tukey's HSD analysis showed that the MDLT population exhibited higher foliar δ^13^C than the XLHT population, which exhibited higher foliar δ^13^C than all others. Significant differences in foliar δ^13^C were also observed between field collected and transplanted specimens, for each of the five populations (MDLT: *F*_1,6_ = 194.6, *P* < 0.001; XLHT: *F*_1,6_ = 16.46, *P* < 0.01; MDMC: *F*_1,13_ = 57.65, *P* < 0.01; XWQ: *F*_1,6_ = 19.23, *P* < 0.01 and HH: *F*_1,5_ = 33.27, *P* < 0.01).

## Discussion

It has recently been suggested that phenotypic plasticity may be the primary adaptive strategy for clonal plant species surviving in a wide range of habitats (Parker *et al*. 2003; Riis *et al*. 2010). Our experimental evidence suggests that this may indeed be the case for *L. chinensis*. Based on foliar δ^13^C, we have provided evidence of local specialization in *L. chinensis* WUE; however, WUE plasticity was more important to allow this clonal plant to occupy a wide range of habitats in the Mongolian steppe.

Plasticity in *L. chinensis* WUE could allow the clonal plant to thrive in the majority of habitats across its distribution. Our study demonstrated that foliar δ^13^C values of *L. chinensis* decreased with increasing precipitation, both in the field investigation and controlled watering experiments (Fig. 1). It means that the clonal plant *L. chinensis* favours higher WUE in drier environment. This negative relationship between plant foliar δ^13^C and precipitation is consistent with the findings of several previous studies (Diefendorf *et al*. 2010; Prentice *et al*. 2011; Wang *et al*. 2013; Liu *et al*. 2015), and indicated that this clonal plant has the ability to respond to changing water conditions by altering its WUE (i.e. plasticity). The study area investigated during the present study covered the majority of *L. chinensis*' distribution in the Mongolian steppe, and we can, therefore, assume that the range of foliar δ^13^C determined by our field investigation (−4.83 ‰) is representative of the natural variation in foliar δ^13^C apparent *in situ*. During the controlled watering experiment, precipitation was applied at two sites, and we can assume that changes in foliar δ^13^C were solely due to the plasticity of each population at each site. The change in foliar δ^13^C observed during the controlled watering experiment (−3.69 ‰) was lower than, but comparable to, variation in foliar δ^13^C observed across different field conditions in the Mongolian steppe; the difference between these two values was −1.14 ‰. Given these data, we can conclude that WUE plasticity allows *L. chinensis* to thrive across most habitats, though there are some differences in foliar δ^13^C of the clonal plant that cannot be explained by plasticity.

Based on the coefficient of determination (i.e. *R*^2^), the strength of the relationship between *L. chinensis* foliar δ^13^C and precipitation was shown to be weaker in the field investigation than during the controlled watering experiments (Fig. 1). This indicated that other factors might affect the relationship between foliar δ^13^C and precipitation across the *in situ* distribution of *L. chinensis*. One likely explanation is the contribution of genetic variation to differences in foliar δ^13^C (Kohorn *et al*. 1994; Lauteri *et al*. 2004; Corcuera *et al*. 2010), although other environmental parameters, such as temperature, light, CO_2_ and altitude, can also affect the foliar δ^13^C of plants (Körner *et al*. 1988; Tazoe *et al*. 2011; Cernusak *et al*. 2013; Wang *et al*. 2013; Liu *et al*. 2015). During the controlled water experiments of the present study, differential plasticity in foliar δ^13^C responses to precipitation was demonstrated for the two *L. chinensis* populations growing in their native habitats. Whereas differences in plasticity could be caused by other environmental parameters, and thus our results do not conclusively demonstrate differential specialization in WUE between these two populations, the results of our transplant experiment confirm that this is the case.

Given that several studies have indicated that local adaptation allows plants to successfully occupy different habitats (Ramirez-Valiente *et al*. 2010; Grassein *et al*. 2014; Si *et al*. 2014), local adaptation could also contribute to the wide distribution of the clonal plant *L. chinensis*. During our transplant experiment, different populations of *L. chinensis* were transplanted into the same environment to identify whether variations in foliar δ^13^C across different populations were caused by local adaptation. In this study, significant differences in foliar δ^13^C were observed between different populations grown for 2 years in the same habitat (Fig. 2). The mean foliar δ^13^C difference between MDMC and XWQ populations was −0.91 ‰, highly comparable to the unaccountable fluctuation in foliar δ^13^C (−1.14 ‰) observed under natural conditions *in situ*. This suggests that variations in WUE observed during the field investigation in the Mongolian steppe may also partially be accounted for by local adaptation. However, our data highlight that the contribution of local adaptation is small relative to plasticity (Fig. 2). In 2011, we investigated the foliar δ^13^C of the same five populations growing in their original collection habitats. Comparison of foliar δ^13^C between each transplanted and *in situ* population revealed a significant difference between the two growth conditions for all populations (Fig. 2). No significant differences in foliar δ^13^C were observed between MDMC and XWQ populations *in situ*, whereas significant variations were apparent after 2 years of growth under transplant conditions (Fig. 2). We also identified significant differences between MDLT, XLHT and HH *in situ* populations, whereas these populations demonstrated no difference during transplant experiments (Fig. 2). Taken together, these findings indicated that plasticity might be more important than local adaptation in affecting *L. chinensis* WUE across its distribution.

## Conclusions

In summary, plasticity causes a greater divergence in *L. chinensis* foliar δ^13^C than local adaptation, although local specialization in foliar δ^13^C exists. Thus, WUE plasticity might be more important than local adaptation in allowing the clonal plant *L. chinensis* to occupy a wide range of habitats in the Mongolian steppe. In order to make a stronger inference that phenotypic plasticity is more important than local adaptation to allow the clonal plant widely distribute in the steppe, more studies focussing on other functional traits or plant fitness are needed in the future.

## Sources of Funding

This work was funded by the National Natural Science Foundation of China (40871032 and 41071209) and the Bureau of Science and Technology for Resources and Environment, Chinese Academy of Sciences (KZCX2-EW-QN604). Y.L. was funded by a scholarship from the China Scholarship Council.

## Contributions by the Authors

All authors together conceived and designed the experiment. Y.L. and L.Z. performed the experiment. Y.L. analysed the data. Y.L. and H.N. wrote the manuscript with comments and help from the other authors.

## Conflict of Interest Statement

None declared.

## Supporting Information

The following additional information is available in the online version of this article –

**Table S1.** Characteristics of all sites used in this study.

Additional Information
