# Supplementary material for: Understanding the wide geographic range of a clonal perennial grass: plasticity versus local adaptation
Source: AoB Plants. 2015 Dec 7;8:plv141. doi: 10.1093/aobpla/plv141 (PMC4705351; doi:10.1093/aobpla/plv141)
Supplement: Additional Information [file supp_8_plv141_index.html]

Understanding the wide geographic range of a clonal perennial grass: plasticity versus local adaptation — Understanding the wide geographic range of a clonal perennial grass: plasticity versus local adaptation — Additional Information 

# Understanding the wide geographic range of a clonal perennial grass: plasticity versus local adaptation

## Additional Information

Additional Information

- Additional Information - Docx file
