# Supplementary material for: Understanding the wide geographic range of a clonal perennial grass: plasticity versus local adaptation
Source: AoB Plants. 2015 Dec 7;8:plv141. doi: 10.1093/aobpla/plv141 (PMC4705351; doi:10.1093/aobpla/plv141)
Supplement: Additional Information [file supp_plv141_plv141supp.docx]

**Supporting information**

Table S1 Characteristics of all sites used in this study

| **Number** | **Latitude (^o^)** | **Longitude (^o^)** | **Altitude (m)** | **Mean annual precipitation (mm)** | **Experiment year** | **Expriment type** | **Experiment region** |
| --- | --- | --- | --- | --- | --- | --- | --- |
| T1 | 46.10 | 106.37 | 1446 | 191.94 | 2008 | Transect | Mongolia |
| T2 | 46.73 | 106.52 | 1411 | 211.40 | 2008 | Transect | Mongolia |
| T3 | 47.24 | 106.65 | 1526 | 229.61 | 2008 | Transect | Mongolia |
| T4 | 47.81 | 106.75 | 1357 | 337.38 | 2008 | Transect | Mongolia |
| T5 | 47.74 | 106.88 | 1446 | 276.90 | 2008 | Transect | Mongolia |
| T6 | 47.81 | 107.03 | 1494 | 337.38 | 2008 | Transect | Mongolia |
| T7 | 47.74 | 108.61 | 1552 | 268.76 | 2008 | Transect | Mongolia |
| T8 | 47.59 | 109.29 | 1550 | 268.81 | 2008 | Transect | Mongolia |
| T9 | 47.53 | 110.83 | 1070 | 260.92 | 2008 | Transect | Mongolia |
| T10 | 47.98 | 111.50 | 1062 | 294.29 | 2008 | Transect | Mongolia |
| T11 | 48.16 | 111.62 | 1092 | 294.29 | 2008 | Transect | Mongolia |
| T12 | 48.59 | 111.85 | 1067 | 321.97 | 2008 | Transect | Mongolia |
| T13 | 48.91 | 112.27 | 964 | 338.55 | 2008 | Transect | Mongolia |
| T14 | 48.76 | 112.93 | 925 | 325.83 | 2008 | Transect | Mongolia |
| T15 | 48.55 | 113.32 | 845 | 283.34 | 2008 | Transect | Mongolia |
| T16 | 46.84 | 113.36 | 1107 | 232.57 | 2008 | Transect | Mongolia |
| T17 | 46.65 | 113.46 | 945 | 211.81 | 2008 | Transect | Mongolia |
| T18 | 47.19 | 113.65 | 941 | 232.57 | 2008 | Transect | Mongolia |
| T19 | 46.18 | 113.85 | 1167 | 209.13 | 2008 | Transect | Mongolia |
| T20 | 48.27 | 113.86 | 814 | 277.94 | 2008 | Transect | Mongolia |
| T21 | 47.62 | 114.17 | 1004 | 236.11 | 2008 | Transect | Mongolia |
| T22 | 47.95 | 114.41 | 786 | 235.41 | 2008 | Transect | Mongolia |
| T23 | 45.94 | 114.43 | 1075 | 218.05 | 2008 | Transect | Mongolia |
| T24 | 45.62 | 114.64 | 1314 | 229.07 | 2008 | Transect | Mongolia |
| T25 | 43.72 | 113.53 | 1027 | 215.13 | 2011 | Transect | Inner Mongolia |
| T26 | 43.88 | 114.24 | 1027 | 228.86 | 2011 | Transect | Inner Mongolia |
| T27 | 44.00 | 115.07 | 1160 | 262.03 | 2011 | Transect | Inner Mongolia |
| T28 | 44.02 | 116.21 | 1059 | 277.04 | 2011 | Transect | Inner Mongolia |
| T29 | 48.64 | 116.82 | 553 | 275.92 | 2011 | Transect | Inner Mongolia |
| T30 | 44.48 | 117.27 | 1055 | 313.15 | 2011 | Transect | Inner Mongolia |
| T31 | 48.45 | 117.31 | 624 | 273.64 | 2011 | Transect | Inner Mongolia |
| T32 | 48.45 | 117.31 | 624 | 273.64 | 2011 | Transect | Inner Mongolia |
| T33 | 44.01 | 117.76 | 1251 | 362.88 | 2011 | Transect | Inner Mongolia |
| T34 | 48.77 | 117.83 | 550 | 292.61 | 2011 | Transect | Inner Mongolia |
| T35 | 49.43 | 118.80 | 616 | 340.80 | 2011 | Transect | Inner Mongolia |
| T36 | 49.43 | 118.80 | 616 | 340.80 | 2011 | Transect | Inner Mongolia |
| T37 | 47.84 | 118.92 | 757 | 313.15 | 2011 | Transect | Inner Mongolia |
| T38 | 47.84 | 118.92 | 757 | 313.15 | 2011 | Transect | Inner Mongolia |
| T39 | 47.66 | 119.29 | 871 | 350.69 | 2011 | Transect | Inner Mongolia |
| T40 | 48.78 | 119.46 | 580 | 341.01 | 2011 | Transect | Inner Mongolia |
| T41 | 44.26 | 120.44 | 381 | 361.01 | 2011 | Transect | Inner Mongolia |
| T42 | 44.17 | 121.73 | 193 | 398.31 | 2011 | Transect | Inner Mongolia |
| MDLT | 43.72 | 113.53 | 1027 | 215.13 | 2009 - 2011 | Transplanting experiment | Inner Mongolia |
| XLHT | 44.02 | 116.21 | 1059 | 277.04 | 2009 - 2011 | Transplanting experiment | Inner Mongolia |
| MDMC | 44.18 | 116.46 | 1087 | 277.04 | 2009 - 2011 | Transplanting experiment | Inner Mongolia |
| XWQ | 44.48 | 117.27 | 1055 | 313.15 | 2009 - 2011 | Transplanting experiment | Inner Mongolia |
| HH | 48.78 | 119.46 | 580 | 341.01 | 2009 - 2011 | Transplanting experiment | Inner Mongolia |
| MDLT | 43.72 | 113.53 | 1027 | 215.13 | 2011 | Watering experiment | Inner Mongolia |
| ABGQ | 43.99 | 115.07 | 1160 | 263.06 | 2011 | Watering experiment | Inner Mongolia |
